# A Clinical Scoring Model to Predict the Effect of Induction Chemotherapy With Definitive Concurrent Chemoradiotherapy on Esophageal Squamous Cell Carcinoma Prognosis

**DOI:** 10.3389/fonc.2021.703074

**Published:** 2021-11-29

**Authors:** Yang Li, Qingwu Du, Xiaoying Wei, Zhoubo Guo, Tongda Lei, Yanqi Li, Dong Han, Xiaoyue Wu, Kunning Zhang, Tian Zhang, Xi Chen, Jie Dong, Baozhong Zhang, Hui Wei, Wencheng Zhang, Qingsong Pang, Ping Wang

**Affiliations:** ^1^ Department of Radiation Oncology, Tianjin Medical University Cancer Institute and Hospital, National Clinical Research Center for Cancer, Key Laboratory of Cancer Prevention and Therapy, Tianjin’s Clinical Research Center for Cancer, Tianjin, China; ^2^ Department of Nutrition Therapy, Tianjin Medical University Cancer Institute and Hospital, National Clinical Research Center for Cancer, Key Laboratory of Cancer Prevention and Therapy, Tianjin’s Clinical Research Center for Cancer, Tianjin, China

**Keywords:** esophageal squamous cell carcinoma, induction chemotherapy, definitive concurrent chemoradiotherapy, progression-free survival, overall survival

## Abstract

**Purpose:**

The aim of the study was to compare the clinical outcomes of induction chemotherapy (IC) followed by definitive concurrent chemoradiotherapy (dCCRT) *versus* chemoradiotherapy alone in patients with esophageal squamous cell carcinoma (ESCC) on the basis of a clinical scoring model.

**Methods:**

A retrospective review of 599 patients with ESCC treated with dCCRT at our institution from 2010 to 2019 was conducted. The patients were divided into two groups based on whether they received IC. A clinical scoring model was performed using the significant variables obtained from the multivariate analysis. The PFS and OS rates were estimated using the Kaplan–Meier method.

**Results:**

During the study period, 182 patients receiving IC followed by dCCRT and 417 dCCRT alone were identified. No significant differences in the PFS and OS rates were observed between the IC group (P=0.532) and the non-IC group (P=0.078). A clinical scoring model was constructed based on independent prognostic factors with scores ranging from 0 to 10.4. The patients were divided into high- and low-risk groups by using the median score as the cutoff value. The PFS rate of patients receiving IC was higher than that of patients treated without IC (P=0.034), while there was no improvement in the OS rate (P=0.794) in the high-risk group. No significant differences in the PFS (P=0.207) or OS (P=0.997) rate were found between the two treatment groups in the low-risk group.

**Conclusions:**

The addition of IC followed by dCCRT for patients with ESCC might be associated with better PFS rates based on a clinical scoring model but has no impact on OS rates. Further prospective studies are warranted for the validation of this model.

## Introduction

Esophageal cancer (EC) is the sixth leading cause of cancer-related mortality in males in the United States, with an estimated 19,260 new cases and 15,530 deaths reported in 2021 (1). Definitive concurrent chemoradiotherapy has been the standard treatment for patients with locally advanced inoperable EC or those who refuse surgical resection (2, 3). Clinical outcomes of EC remain unsatisfactory despite recent improvements in treatment modalities and advancements in radiotherapy. It has been reported that the 5-year relative survival rate of patients with EC is as low as 20% when all stages are combined (1). Therefore, there has been increasing focus on improving the survival rate.

Previous studies have proven the long-term benefit of IC and subsequent conversion surgery (CS) in patients with locally advanced EC (4, 5). However, the value of adding IC for patients with EC treated with dCCRT is unclear, especially for those with ESCC, which is the most common histologic type in China (6). The results of a prior study indicated better PFS rates in high-risk patients with EC treated with IC before dCCRT than in those receiving dCCRT alone using recursive partitioning analysis (PRA; a total of 146 patients with ESCC were enrolled in this study) (7). Ku et al. presented a case report of a patient with ESCC with bronchial invasion who was treated with IC followed by dCCRT and achieved a clinical complete response with no evidence of disease 12 months after his initial diagnosis (8). We then hypothesized that IC might improve prognosis by reducing the risk of perforation and might benefit patients with ESCC with certain clinical features. A randomized phase III trial initiated in Japan to compare the OS rates of patients treated with docetaxel plus cisplatin and 5-fluorouracil (DCF) followed by CS or dCCRT and definitive CRT alone for locally advanced inoperable ESCC is currently recruiting patients (9). We are looking forward to the research results.

This study is based on a large cohort of patients with ESCC who received dCCRT that we used to build a clinical scoring model for the identification of patients who may benefit from IC, with the hope of providing advice and guidance for clinicians and patients on appropriate treatment and management strategies.

## Patients and Methods

### Patient Selection

**Figure 1:**
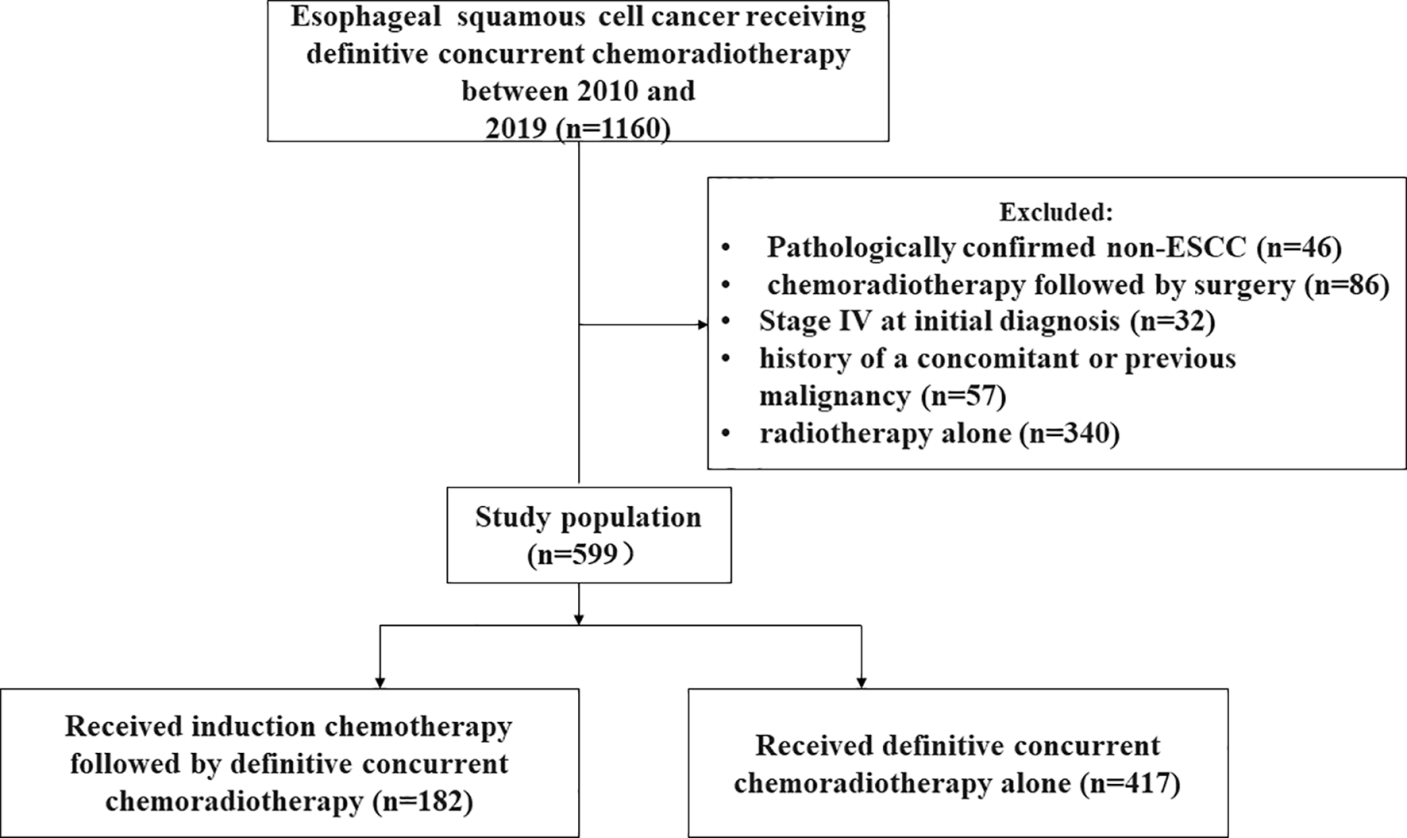
The flowchart of the screening process.

A cohort of 1160 consecutively recruited patients with EC treated by radiotherapy at our institute was retrospectively analyzed from 2010 to 2019. The criteria for inclusion were as follows: 1) pathologically confirmed ESCC; 2) Karnofsky performance status (KPS) score ≥70; 3) no distant organ metastasis; 4) no history of a concomitant or previous malignancy; 5) underwent intensity-modulated radiation therapy (IMRT)-based definitive concurrent chemoradiotherapy; and 6) had unresectable EC or refused surgery. A total of 599 patients with ESCC met the above criteria and were selected for analysis. The flowchart of the screening process was shown in **Figure 1**. The study was approved by the Ethics Committee of Tianjin Medical University Cancer Institute and Hospital. The patients were not required to sign an informed consent form for this retrospective study.

### Treatment

#### Chemotherapy

All patients received concurrent chemotherapy during radiotherapy. Before receiving dCCRT, a proportion of patients were treated with 1 to 6 cycles of IC. The IC regimens were all platinum-based. A large number of our patients received concurrent chemotherapy with a weekly or three-weekly schedule of paclitaxel and platinum-based drugs. For subsequent consolidation chemotherapy, the regimens were selected based on patient age, general physical condition, and physician judgment.

#### Radiotherapy

Radiotherapy used intensity-modulated radiation therapy (IMRT) technology. The gross tumor volume (GTV) was defined as the primary tumor, and the gross tumor volume of nodes (GTVnd) was defined as positive lymph nodes. The clinical target volume (CTV) was defined as the visible GTV, GTVnd and subclinical regions at risk for involvement. The CTV comprised an expansion of 3.0 cm margin in the cephalad and caudal directions and a margin of 0.5 cm around the GTV in the left, right, anterior and posterior directions. The planning gross target volume (PGTV) was obtained by adding an isotropic margin of 0.5 cm to the GTV combined with GTVnd. The planning target volume (PTV) was defined as the CTV plus a 0.5 cm margin in all directions. The prescription dose of 60 Gy in 30 fractions of 2.0 Gy per fraction to the GTV and PGTV and a dose of 54 Gy in 30 fractions of 1.8 Gy per fraction to the CTV and PTV were delivered. Ninety-five percent of PGTV and PTV volumes were covered by the prescribed dose. The dose constraints to organs at risk (OAR) at our institution were as follows: lung V20 ≤30%, V30 ≤20%, and the mean lung dose (MLD) was ≤16 Gy; heart V30 ≤40%, V40 ≤30%, and the mean heart dose (MHD) was ≤28 Gy; and the maximum dose to the spinal cord was <45 Gy.

### Endpoints

The end points included the overall survival (OS) rate, the progression-free survival (PFS) rate and treatment-related toxicity. We defined OS as the time from the first treatment to the date of death from any cause or the date of last follow-up. The PFS rate was calculated from the time of first treatment to disease progression, including local recurrence (LR), regional recurrence (RR), and distant metastasis (DM). Patients who did not experience an event of interest were censored at their last follow-up or the date of death.

### Toxicity and Follow-Up

The toxicities of treatment were evaluated according to the Common Terminology Criteria for Adverse Events (CTCAE, version 4.0). Patients attended regularly scheduled follow-up physical examinations, blood tests, chest and abdominal CT scans, barium esophagography, and ultrasonography at 3-month intervals for the first two years, every 6 months for the next three years, then annually.

### Statistical Analysis

Descriptive statistics were performed to examine the baseline characteristics of all patients. Age, KPS, radiation dose and primary tumor length were categorized with the median value as the cutoff. Grouping by the maximum lymph node diameter was performed as previously described (7). Continuous variables are shown as the means with standard deviation and were compared using the Mann-Whitney U test. Categorical variables were compared by using the chi-square test or Fisher’s exact test. Median follow-up was reported with reverse Kaplan-Meier estimate (10). Kaplan-Meier analysis with a log-rank test was used to compare the differences in the PFS and OS rates between the two groups. We used univariate and multivariate Cox proportional hazard models to examine the influence of different variables on the OS rate. Hazard ratios and their associated 95% confidence intervals (95% CIs) were obtained from the Cox regression analysis. To minimize potentially confounding effects between the two treatment groups, propensity score matching (PSM) was performed using Stata software. Using the OS rate as an endpoint, a clinical scoring model was performed by using the significant variables obtained from the multivariate analysis. The score was the weighted sum of the significant variables of which the weights were defined as the quotient of the corresponding estimated coefficient from a Cox regression analysis divided by the smallest chi-square coefficient (11, 12). All statistical tests were two-sided and P values < 0.05 were considered statistically significant. Analyses were conducted using SPSS v22.0 (IBM SPSS, New York), and figures were created using the Stata software program (version 14.1; StataCorp, College Station, TX, USA).

## Results

### Patient and Tumor Characteristics

A total of 599 eligible patients with ESCC were included in the study, and they were divided into the following two treatment groups: IC followed by dCCRT (IC group, n=182) and dCCRT alone (non-IC group, n=417). The patient clinical and treatment characteristics are summarized in **Table 1**. The median age of the whole cohort at diagnosis was 61 years (ranging from 30 to 86 years old). The sex ratio of males to females was 6:2. The median primary tumor length was 6.0 cm (range, 1.2-18.0 cm).

**Table 1:** Patient characteristics.

Characteristic	Total, N = 599 (%)	IC group, n = 182 (%)	Non-IC group, n = 417 (%)	P
Age, y				0.033
≤60	270 (45.1)	94 (51.6)	176 (42.2)	
>60	329 (54.9)	88 (48.4)	241 (57.8)	
Sex				0.180
Male	516 (86.1)	162 (89.0)	354 (84.9)	
Female	83 (13.9)	20 (11.0)	63 (15.1)	
Smoking history				0.138
Yes	417 (69.6)	135 (74.2)	282 (67.6)	
No	139 (23.2)	39 (21.4)	100 (24.0)	
Unknown	43 (7.2)	8 (4.4)	35 (8.4)	
KPS				<0.001
≥90	395 (65.9)	142 (78.0)	253 (60.7)	
<90	181 (30.2)	40 (22.0)	141 (33.8)	
Unknown	23 (3.8)	0 (0.0)	23 (5.5)	
Weight loss				0.443
Yes	205 (34.2)	56 (30.8)	149 (35.7)	
No	372 (62.1)	120 (65.9)	252 (60.4)	
Unknown	22 (3.7)	6 (3.3)	16 (3.8)	
Pain of chest and back				0.906
Yes	97 (16.2)	29 (15.9)	68 (16.3)	
No	491 (82.0)	149 (81.9)	342 (82.0)	
Unknown	11 (1.8)	4 (2.2)	7 (1.7)	
Tumor location				0.372
Cervical/UT	230 (38.4)	65 (35.7)	165 (39.6)	
MT/LT	369 (61.6)	117 (64.3)	252 (60.4)	
Clinical T stage, 8th				0.400
T1-2	54 (9.0)	16 (8.8)	38 (9.1)	
T3-4	502 (83.8)	149 (81.9)	353 (84.7)	
Tx	43 (7.2)	17 (9.3)	26 (6.2)	
Clinical N stage, 8^th^				0.022
N0	96 (16.0)	22 (12.1)	74 (17.7)	
N1-3	470 (78.5)	144 (79.1)	326 (78.2)	
Nx	33 (5.5)	16 (8.8)	17 (4.1)	
Clinical TNM stage, 8th				<0.001
I	12 (2.0)	0 (0.0)	12 (2.9)	
II	64 (10.7)	18 (9.9)	46 (11.0)	
III	261 (43.6)	62 (34.1)	199 (47.7)	
IV	224 (37.4)	83 (45.6)	141 (33.8)	
Unknown	38 (6.3)	19 (10.4)	19 (4.6)	
Tumor length, cm				0.172
≤6	310 (5.8)	85 (46.7)	225 (54.0)	
>6	257 (42.9)	84 (46.2)	173 (41.5)	
Unknown	32 (5.3)	13 (7.1)	19 (4.6)	
MLND, cm				0.006
<1	285 (47.6)	69 (37.9)	216 (51.8)	
≥1	291 (48.6)	106 (58.2)	185 (44.4)	
Unknown	23 (3.8)	7 (3.8)	16 (3.8)	
Adjuvant chemotherapy				0.004
Yes	238 (39.7)	88 (48.4)	150 (36.0)	
No	361 (60.3)	94 (51.6)	267 (64.0)	
Radiation dose, Gy				0.145
<54	190 (31.7)	51 (28.0)	139 (33.3)	
≥54	390 (65.1)	122 (67.0)	268 (64.3)	
Unknown	19 (3.2)	9 (4.9)	10 (2.4)	

KPS, Karnofsky performance status; UT, Upper thoracic; MT, Middle thoracic; LT, Lower thoracic; MLND, Maximum lymph node diameter.

The median radiation dose was 54 Gy in the whole cohort (range, 41.4-70Gy). In our cohort, 65/599 patients (10.9%) developed esophageal fistula.

The differences in sex, smoking history, weight loss, pain in the chest and back, tumor location, clinical T stage, tumor length, and radiation dose were not statistically significant between the two groups (P > 0.05, **Table 1**). There were more younger patients in the IC group than the non-IC group (P=0.033). Compared with those in the non-IC group, patients in the IC group had a higher ratio of KPS ≥90 (78.0% *vs.* 60.7%, P<0.001), a higher rate of N1-3 (79.1% *vs.* 78.2%, P=0.022), a larger maximum lymph node diameter (58.2% *vs.* 44.4%, P=0.006), and a higher proportion of adjuvant chemotherapy (48.4% *vs.* 36.0%, P=0.004).

### Chemotherapy Regimens

For patients treated with IC, the median number of cycles administered was 2 (range 1–6). A total of 59/182 (32.4%) patients received the three-drug combination IC regimen, including taxane (paclitaxel or docetaxel), platinum (nedaplatin, oxaliplatin or cisplatin), and fluoropyrimidine (5-fluorouacil, tegafu). Two drug treatment regimens were prescribed in 122/182 (67.0%) patients, with 75 patients receiving paclitaxel-platinum combined chemotherapy, 45 patients receiving platinum in combination with fluoropyrimidine, 1 patient receiving gemcitabine and cisplatin, and 1 patient receiving vinorelbine and nedaplatin. The remaining patient received single-agent paclitaxel therapy. The details of the use of IC were shown as **Supplementary Table 1**. The regimens were selected based on patient age, general physical condition, response to chemotherapy, patient willingness, and physician judgment.

For subsequent adjuvant chemotherapy (ACT), a total of 238 patients (39.7%) receiving ACT following dCCRT in our study, the median number of chemotherapy cycles was 2 (range, 1-6). For patients treated with ACT, 51 patients (21.4%) had 1 cycle of ACT, 84 (35.3%) had two cycles, 45 (18.9%) had three cycles, 39 (16.4%) had four cycles, 6(2.5%) had five cycles, and 13(5.5%) had six cycles. A total of 11/238 (4.6%) patients received the three-drug combination of regimens based on platinum (oxaliplatin, cisplatin, nedaplatin) combined with taxane (paclitaxel or docetaxel) and fluoropyrimidine (5-fluorouacil, tegafu). Two ACT regimens were used in 217/238 (91.2%) patients, with 163/217 (75.1%) patients receiving paclitaxel-platinum combined chemotherapy, 50/217(23.0%) patients treated with platinum in combination with fluoropyrimidine, and 4/217(1.8%) patients receiving a combination of irinotecan and fluoropyrimidine. And 10/217(4.2%) patients were treated with single-agent chemotherapy regimen of tegafu.

Tumor response was evaluated in 96 patients (52.7%) after IC. Specifically, complete response (CR) was achieved in 1 patient (1.0%), partial response (PR) in 44 patients (45.8%), stable disease (SD) in 40 patients (41.7%), and progressive disease (PD) in 11 patients (11.5%). Overall, a total of 559 patients (93.3%) had post‐treatment tumor assessments. CR was obtained in 25 patients (4.2%), PR in 362 patients (60.4%), SD in 131 patients (21.9%), and PD in 41 patients (6.8%) for the whole cohort, respectively.

### Toxicity and Dose of OAR

The observed treatment-related toxicities graded using the CTCAE are shown in **Table 2**. The most common acute toxicities were radiation esophagitis and leukocytopenia, occurring in 43.9% and 52.0% of all patients with recorded toxicities. The incidence of hematological toxicities grade ≥2 in the IC group was higher than that in the non-IC group (leukocytopenia, 27.3% vs. 25.1%; anemia, 11.1% vs. 6.9%; thrombocytopenia, 9.3% vs. 6.1%), although the difference was not statistically significant (p=0.085, p= 0.068, p=0.077, respectively). There were no significant differences in the rates of radiation esophagitis, radiation pneumonitis and radiodermatitis between the two groups (P=0.225; P=0.205; P=0.663). No treatment-related deaths or grade 5 toxicities occurred. The comparison of radiation dose to OAR between the two groups is shown in **Table 3**. No differences were observed in lung doses, heart doses or maximum spinal cord doses between the two groups (all p>0.05).

**Table 2:** Comparison of toxicities between the two groups.

Toxicity	IC group [n = 182(%)]				Non-IC group [(n = 417(%)]				
	Grade 1	Grade 2	Grade 3	Grade 4	Grade 1	Grade 2	Grade 3	Grade 4	P
RE*	16 (10.3)	40 (25.6)	8 (5.1)	0 (0)	28 (8.4)	84 (25.1)	35 (10.4)	4 (1.2)	0.255
RP*	7 (4.5)	2 (1.3)	1 (0.6)	0 (0)	30 (9.0)	9 (2.7)	3 (0.9)	3 (0.9)	0.205
Leukocytopenia	52 (32.1)	32 (19.8)	9 (5.6)	3 (1.9)	91 (23.9)	71 (18.7)	23 (6.1)	1 (0.3)	0.085
Neutropenia	31 (19.5)	14 (8.8)	7 (4.4)	1 (0.6)	51 (14.0)	18 (5.0)	12 (3.3)	6 (1.7)	0.120
Anemia	56 (34.6)	17 (10.5)	1 (0.6)	0 (0)	99 (27.1)	24 (6.6)	1 (0.3)	0 (0)	0.068
Thrombocytopenia	29 (17.9)	10 (6.2)	3 (1.9)	2 (1.2)	48 (13.2)	14 (3.9)	8 (2.2)	0 (0)	0.077
radiodermatitis	12 (6.9)	9 (5.2)	2 (1.1)	0 (0)	29 (7.3)	17 (4.3)	1 (0.3)	2 (0.5)	0.663

RE, Radiation esophagitis; RP, Radiation pneumonitis.

**Table 3:** Comparison of radiation dose to OARs between the two groups.

Variables	IC group (n = 182)	Non-IC group (n = 417)	P
Mean lung dose (Gy)	10.56 ± 3.05	10.36 ± 5.05	0.699
Lung V5 (%)	42.14 ± 17.05	38.75 ± 17.82	0.110
Lung V20 (%)	18.36 ± 10.68	17.50 ± 10.30	0.472
Lung V30 (%)	10.59 ± 6.51	10.39 ± 7.59	0.813
Mean heart dose (Gy)	17.77 ± 11.10	15.69 ± 12.01	0.168
Heart V30 (%)	22.99 ± 18.83	19.18 ± 19.22	0.095
Heart V40 (%)	12.51 ± 11.30	11.11 ± 12.08	0.333
Maximum spinal cord dose (Gy)	39.12 ± 7.28	40.78 ± 13.71	0.235

V5, Volumes receiving more than 5 Gy; V20, Volumes receiving more than 20 Gy; V30, Volumes receiving more than 30 Gy; V40, Volumes receiving more than 40 Gy.

### Recurrence and Survival

The median follow-up period was 33 months for the entire cohort. In total, 339 (56.6%) patients experienced disease recurrence, and 301 (50.3%) died over the course of follow-up. Distant metastases occurred in 151 patients (25.2%) and the metastatic sites included the lung, bone, liver, brain, pleura, kidney, adrenal gland, lymph nodes and soft tissue. Of these patients, 122 developed distant metastases in a single organ: 66 lung metastases, 20 liver metastases, 19 bone metastases, 8 soft tissue metastases,4 brain metastases, 3 pleura metastases, 1 kidney metastases, and 1 adrenal gland metastases and 29 had multi-site metastases. Ninety-two patients in the IC group experienced recurrence, of which 54 (29.7%) experienced local recurrence (LR), 44 (24.2%) experienced regional recurrence (RR), and 43 (23.6%) experienced distant metastases (DM). In the non-IC group, a total of 247 (59.2%) patients experienced relapse, of which 153 (36.7%) experienced LR, 105 (25.2%) experienced RR and 108 (25.9%) experienced DM. There were no significant differences in the LR, RR or DM rates between the two groups (P=0.097, P=0.794, P=0.556, respectively).

**Figure 2:**
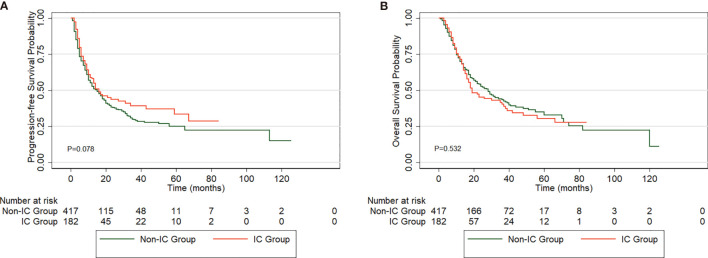
Comparison of PFS **(A)** and OS **(B)** in esophageal squamous cell carcinoma (ESCC) patients between the two groups.

The median PFS and OS rates for the entire group of patients were 15 months (95% CI, 12.4-17.6) and 25 months (95% CI, 19.7-30.3), respectively. The 1-, 3-, and 5-year PFS rates were 58.1%, 39.3%, and 33.4% in the IC group and 52.2%, 29.1%, and 24.7% in the non-IC group, respectively (P=0.078) (**Figure 2A**). No significant difference in OS was observed between the IC group (1-year OS, 71.4%; 3-year OS, 40.3%; 5-year OS, 30.3%) and the non-IC group (1-year OS, 69.9%; 3-year OS, 43.5%; 5-year OS, 32.9%) (P=0.532) (**Figure 2B**).

### Univariate and Multivariate Analysis

We used univariate and multivariate Cox proportional hazard regressions to analyze prognostic factors influencing the OS rate in patients with ESCC (**Table 4**). The univariate analysis revealed that the OS rate was significantly associated with the following factors: weight loss (p=0.038), tumor location (p=0.002), clinical T stage (p=0.010), clinical N stage (p<0.001), tumor length (p<0.001), maximum lymph node diameter (p<0.001) and adjuvant chemotherapy (p=0.004). Further multivariate analysis showed that clinical T stage (P=0.029), tumor length (p=0.004), maximum lymph node diameter (p<0.001) and adjuvant chemotherapy (p=0.007) were independent factors affecting patient survival (**Table 4**).

**Table 4:** Univariate and multivariate analysis of clinical and treatment factors related to overall survival (N=599).

Factor	Comparison	Univariate		Multivariate		
		P	χ2	HR (95%CI)	P	Score
Age, y	≤60 *vs.* >60	0.976				
Sex	Male *vs.* Female	0.146				
Smoking history	Yes *vs.* No	0.162				
KPS	≥90 *vs.* <90	0.179				
Weight loss	Yes *vs.* No	0.038				
Pain of chest and back	Yes *vs.* No	0.850				
Tumor location	Cervical/UT *vs.* MT/LT	0.002				
Clinical T stage, 8th	T1-2 *vs.* T3-4	0.010	4.781	1.872 (1.067-3.283)	0.029	1.0 *vs.* 0
Clinical N stage, 8th	N0 *vs.* N1-3	<0.001				
Tumor length, cm	>6 *vs.* ≤6	<0.001	8.404	1.465 (1.132-1.897)	0.004	1.8 *vs.* 0
MLND, cm	≥1 *vs.* <1	<0.001	29.211	2.091 (1.600-2.733)	<0.001	6.1 *vs.* 0
ACT	Yes *vs.* No	0.001	7.273	0.696 (0.535-0.906)	0.007	1.5 *vs.* 0
Radiation dose, Gy	<54 *vs.* ≥54	0.136				

HR, Hazard Ratio; UT, Upper thoracic; MT, Middle thoracic; LT, Lower thoracic; MLND, Maximum lymph node diameter; ACT, Adjuvant chemotherapy.

### Clinical Scoring Model

**Figure 3:**
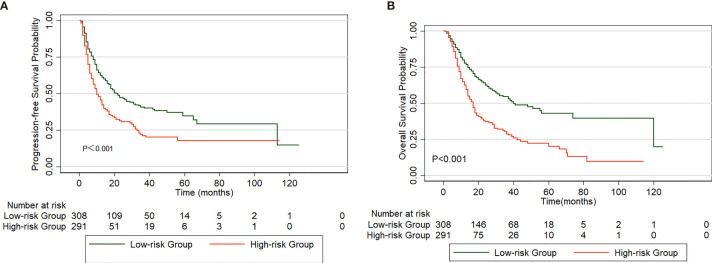
Comparison of PFS **(A)** and OS **(B)** in patients divided into high and low-risk group based on a clinical scoring model.

The parameters with statistical significance in the multivariate analysis are shown in **Table 4**. The score of each parameter is weighted according to its relative contribution determined by the chi-square score. Specifically, the score for each variable was calculated by dividing its chi-square value obtained from the Cox regression analysis by the smallest chi-square coefficient. The score ranged from 0 to 10.4 after adding up these variables. Using the median value as a cutoff point, we then separated patients into high (>5.5) and low (≤5.5) risk groups. We evaluated the PFS rates of the high-risk group (n=291) and low-risk group (n=308), and the PFS rate of the high-risk group was significantly shorter than that of the control group (p<0.001, **Figure 3A**). Moreover, patients in the high-risk group had worse OS rates than those in the low-risk group (p<0.001, **Figure 3B**).

### Prognostic Analysis in Risk Groups

**Figure 4:**
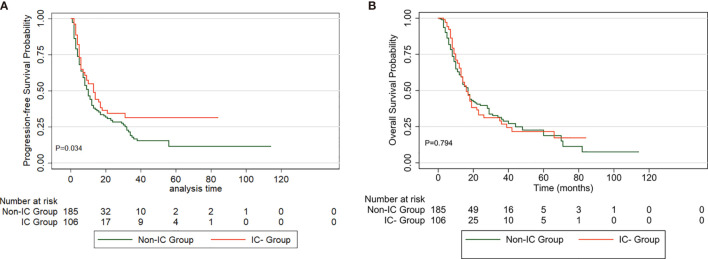
PFS **(A)** and OS **(B)** curve for patients treated with induction chemotherapy (IC) *versus* non-IC in high-risk group.

The baseline clinical and treatment characteristics of the high-risk group of 291 patients are shown in **Table 5**. A total of 106 patients received IC followed by dCCRT, and 185 underwent dCCRT alone. The baseline characteristics were similar between the two groups, with no significant differences. The PFS rate of patients receiving IC was comparatively higher than that of patients who did not receive IC (P=0.034, **Figure 4A**). The prognosis of the IC group, with a 5-year OS of 21.6%, was comparable to that of the non-IC group, with a 5-year OS of 18.8% (P=0.794, **Figure 4B**).

**Table 5:** Comparison of demographic and therapeutic characteristics of IC and non-IC groups in high-risk patients.

Characteristic	Total, N = 291 (%)	IC group, n = 106 (%)	Non-IC group, n = 185 (%)	P
Age, y				0.163
≤60	149 (51.2)	60 (56.6)	89 (48.1)	
>60	142 (48.8)	46 (43.4)	96 (51.9)	
Sex				0.525
Male	262 (90.0)	97 (91.5)	165 (89.2)	
Female	29 (10.0)	9 (8.5)	20 (10.8)	
Smoking history				0.954
Yes	216 (77.7)	81 (77.9)	135 (77.6)	
No	62 (22.3)	23 (22.1)	39 (22.4)	
KPS				0.052
≥90	214 (73.5)	85 (80.2)	129 (69.7)	
<90	77 (26.5)	21 (19.8)	56 (30.3)	
Weight loss				0.307
Yes	99 (35.2)	32 (31.4)	67 (37.4)	
No	182 (64.8)	70 (68.6)	112 (62.6)	
Pain of chest and back				0.522
Yes	53 (18.5)	17 (16.5)	36 (19.6)	
No	234 (81.5)	14986 (83.5)	148 (80.4)	
Tumor location				0.730
Cervical/UT	97 (33.3)	34 (32.1)	63 (34.1)	
MT/LT	194 (66.7)	72 (67.9)	122 (65.9)	
Clinical T stage, 8th				0.489
T1-2	21 (7.7)	9 (9.2)	12 (6.9)	
T3-4	252 (92.3)	89 (90.8)	163 (93.1)	
Clinical N stage, 8th				0.455
N0	1 (0.4)	0 (0.0)	1 (0.6)	
N1-3	279 (99.6)	100 (100.0)	179 (99.4)	
Clinical TNM stage, 8th				0.118
I	1 (0.4)	0 (0.0)	1 (0).6	
II	8 (2,9)	3 (3.9)	5 (2.8)	
III	129 (46.4)	37 (37.4)	92 (51.4)	
IV	140 (50.4)	59 (59.6)	81 (45.3)	
Tumor length, cm				0.779
≤6	141 (50.9)	51 (52.0)	90 (50.3)	
>6	136 (49.1)	47 (48.0)	89 (49.7)	
MLND, cm				–
<1	0 (0.0)	0 (0.0)	0 (0.0)	
≥1	291 (100.0)	106 (100.0)	185 (100.0)	
Adjuvant chemotherapy				0.135
Yes	118 (40.5)	49 (46.2)	69 (37.3)	
No	173 (59.5)	57 (53.8)	116 (62.7)	
Radiation dose, Gy				0.609
<54	88 (31.1)	33 (33.0)	55 (30.1)	
≥54	195 (68.9)	67 (67.0)	128 (69.9)	

**Figure 5:**
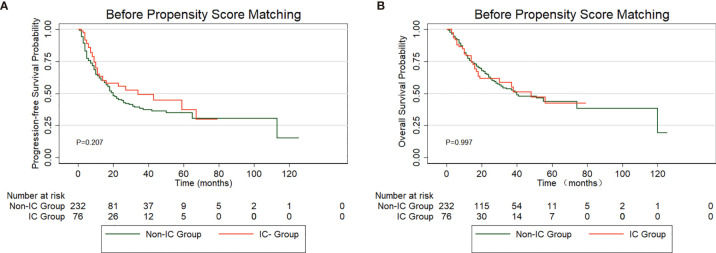
PFS **(A)** and OS **(B)** curve for patients treated with induction chemotherapy (IC) *versus* non-IC in low-risk group before propensity score matching (PSM).

**Figure 6:**
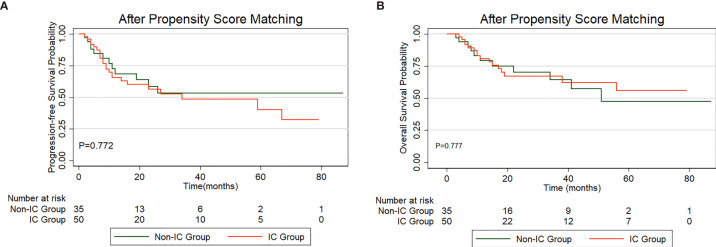
PFS **(A)** and OS **(B)** curve for patients treated with induction chemotherapy (IC) *versus* non-IC in low-risk group after propensity score matching (PSM).

In total, 308 patients in the low-risk group received IC (IC group), and 232 patients did not receive IC (non-IC group), as shown in **Supplementary Table 2**. Kaplan-Meier analysis of the PFS and OS rates revealed no significant difference between the groups (PFS: P=0.207, **Figure 5A**; OS: P=0.997, **Figure 5B**). The baseline characteristics were balanced between the two groups using propensity score matching (PSM) (**Supplementary Table 2**). Fifty patients who received IC were matched with thirty-five patients without IC treatment by PSM analysis. Likewise, no significant differences were found between the two treatment groups in the PFS (P=0.772) or OS rates (P=0.777) (**Figures 6A, B**). Furthermore, In low-risk group, we found patients showed a longer OS treated with ACT (P=0.003), while no significant difference in PFS (P=0.224). The survival analysis revealed that ACT affected the OS of patients (P=0.049) but had no significant impact on PFS (P=0.261) in high-risk group.

### High-Risk Group Analysis by IC Regimen

Because the wide variation in the IC regimen, we then investigated whether the number of chemotherapy combination and the regimens would have an effect on PFS in patients with high-risk group. In patients who received two drug treatment regimens (66/291, 22.7%) or three drug combination regimens (39/291, 13.4%), no significant difference in PFS (P=0.259) between two groups was observed. In addition, for 40 high-risk patients treated with taxane in combination with platinum and 25 patients receiving platinum-fluoropyrimidine combined chemotherapy regimens, similarly, the PFS did not differ for the two different regimens (P=0.591).

## Discussion

The prognostic effect of IC on patients with ESCC receiving dCCRT is unclear.

In this retrospective study, we used a clinical scoring model composed of readily available clinical parameters to divide patients with ESCC into high-risk and low-risk groups, with significant differences in the PFS and OS rates. The results showed that the PFS rate of patients who received IC was longer than that of patients without IC in the high-risk group. However, no significant differences were observed in the PFS and OS rates between the IC group and the non-IC group in the low-risk group. The scoring model might be effective for predicting prognosis and distinguishing patients who could benefit most from IC to provide a reference for clinicians.

The role of IC before dCCRT for EC has long been debated. Berger B et al. analyzed 129 patients with locally advanced EC and found no significant difference between the definitive chemoradiation (CRT) group and the IC followed by CRT with or without surgery (C-CRT/S) group (13). IC versus no IC followed by preoperative chemoradiation was evaluated in a phase II randomized clinical trial conducted by Ajani et al. in patients with EC and the results indicated that IC did not significantly increase the pCR rate or prolong the OS rate (14). Chen MQ et al. included 60 patients with ESCC treated with definitive CRT and concluded that IC treatment using the current regimen does not prolong the overall survival, locoregional failure-free survival or distant failure-free survival rates (15). However, in a retrospective analysis of 496 patients with EC, Xi M et al. found that patients in the high-risk group who received IC had better PFS and locoregional failure-free survival rates than non-IC patients, while there were no significant differences between the 2 treatment groups in low-risk or intermediate-risk patients. Luo et al. reported that the addition of IC for locally advanced ESCC in 267 patients showed (7) better median OS rates in a matched case-control study (16). Overall, recent studies have shown that the addition of IC for high-risk or locally advanced EC might improve prognosis, which is consistent with the main findings of our study in which patients with ECSS treated with IC followed by dCCRT in the high-risk group tended to have longer a PFS rate than those without IC. The reason might be that IC was associated with a lower risk of fistula formation and might also downstage the tumor and improve dysphagia (8, 17–19), thus improving the prognosis. IC might control minor metastases to contribute to the survival benefit (20).

The results of our study demonstrated that a maximum lymph node diameter (MLND) ≥1 cm was associated with poor survival and had the greatest predictive value among the four prognostic factors affecting survival in our clinical scoring model. A previous study reported by Dhar et al. showed that patient survival decreased with each millimeter increment in MLN size and that MLNs <1 cm were associated with significantly better OS rates (21). A study using recursive partitioning analysis (RPA) indicated that a maximum metastatic lymph node diameter (ND) ≥2.8 cm was associated with the worst prognosis (22). Recently, Zhao et al. reviewed 376 patients with ESCC treated with definitive (chemo-) radiotherapy and revealed that the larger the lymph node size, the worse the prognosis (23). Although the cutoff value of the maximum diameter of the metastatic lymph node varied in different studies, MLND has consistently been found to be an important prognostic factor of EC. We also found that tumor length and clinical T stage were important factors for prognosis, which has been reported in many previous studies (7, 22, 24–26).

Interestingly, in our study, we found that receiving adjuvant chemotherapy (ACT) following dCCRT was a significantly favorable factor for OS, which was controversial for EC receiving concurrent chemoradiotherapy. Current studies have suggested that ACT does not improve the OS rate of patients with ESCC treated with dCCRT (27, 28). Wu et al. reported that ACT remained a favorable prognostic factor for OS in patients with ESCC, while a propensity score analysis failed to show an additional OS benefit with ACT (29). Koh et al. reviewed 73 patients with ESCC who underwent dCCRT, and ACT was administered in 56 patients. The author concluded that the addition of chemotherapy after dCCRT improves the OS rate (30). However, the small sample size limits the reliability of the results. The rate of KPS ≥90 in patients receiving ACT following dCCRT was higher than that in patients without ACT in our study (74.4% *vs.* 60.4%, P<0.001). In addition, stage III and IV were more frequently observed in patients without ACT (79.0% *vs.* 82.3%, P<0.001). These observations might explain why patients treated with ACT after dCCRT had better OS rates in our study. Future prospective studies are needed to confirm this association.

The influence of therapeutic toxicity cannot be ignored. Previous studies have demonstrated that the addition of IC has a manageable toxicity profile and the most common toxicities are hematological, including leukopenia and neutropenia (4, 7, 16, 17, 20, 31). These results were in accordance with our findings. However, Wang et al. reviewed 23 patients with EC treated with definitive chemoradiotherapy from 2000 to 2003 and concluded that IC before dCCRT was significantly associated with an increased risk of grade ≥2 pneumonitis, which was inconsistent with our findings as we did not observe an increase in radiation pneumonitis risk (7, 16). One possible reason for this difference might be the small sample size of the study conducted by Wang et al. Collectively, our stud studies indicate that toxicities were not increased by the addition of IC.

There are some limitations to our research. First, this study was retrospective, so the potential for unmeasured patient-, disease-, and institution-associated confounding factors that may contribute to prognosis must be considered. Second, the different IC regimens and the number of IC cycles might affect the prognosis to some extent. Finally, information on the relapse or death of some patients who were not reexamined regularly after discharge was obtained through telephone follow-up, which might have led to inaccurate information about recurrence in individual patients.

## Conclusions

Our study demonstrated that IC followed by dCCRT might be a promising treatment strategy to provide a better PFS rate in high-risk patients with ESCC based on a clinical scoring model. Clearly, further multiple-center prospective studies are required to verify our conclusion.

## Data Availability Statement

The original contributions presented in the study are included in the article/**Supplementary Material**. Further inquiries can be directed to the corresponding authors.

## Ethics Statement

The study was approved by the Ethics Committee of Tianjin Medical University Cancer Institute and Hospital. The patients were not required to sign an informed consent form for this retrospective study.

## Author Contributions

Conception and design: PW, QP, WZ. Administrative support: PW, QP, WZ. Provision of study materials or patients: QP, WZ, PW. Collection and assembly of data: YangL, QD, XWe. Data analysis and interpretation: YangL, QD. Manuscript writing: All authors. Final approval of manuscript: All authors. All authors contributed to the article and approved the submitted version.

## Funding

This work was supported by grants from the Chinese National Key Research and Development Project [No. 2018YFC1315601].

## Conflict of Interest

The authors declare that the research was conducted in the absence of any commercial or financial relationships that could be construed as a potential conflict of interest.

## Publisher’s Note

All claims expressed in this article are solely those of the authors and do not necessarily represent those of their affiliated organizations, or those of the publisher, the editors and the reviewers. Any product that may be evaluated in this article, or claim that may be made by its manufacturer, is not guaranteed or endorsed by the publisher.

## Abbreviations

IC, Induction chemotherapy; dCCRT, definitive concurrent chemoradiotherapy; ESCC, esophageal squamous cell carcinoma; EC, esophageal cancer; CS, conversion surgery; PRA, recursive partitioning analysis; KPS, karnofsky performance status; IMRT, intensity-modulated radiation therapy; GTV, gross tumor volume; GTVnd, gross tumor volume of positive nodes; PGTV, planning gross target volume; PTV, planning target volume; OAR, organs at risk; MLD, mean lung dose; MHD, mean heart dose; OS, overall survival; PFS,progression-free survival; LR, local recurrence; RR, regional recurrence; DM, distant metastasis; CTCAE, Common Terminology Criteria for Adverse Events; PSM, propensity score matching.
