# Supplementary material for: A Clinical Scoring Model to Predict the Effect of Induction Chemotherapy With Definitive Concurrent Chemoradiotherapy on Esophageal Squamous Cell Carcinoma Prognosis
Source: Front Oncol. 2021 Nov 29;11:703074. doi: 10.3389/fonc.2021.703074 (PMC8666950; doi:10.3389/fonc.2021.703074)
Supplement: Supplementary file 1 [file Table_1.docx]

Supplementary Table 1. The details of induction chemotherapy used in ESCC patients

|  | Number (n=182) | % |
| --- | --- | --- |
| IC cycles |  |  |
| 1-2 | 141 | 77.5 |
| 3 | 27 | 14.8 |
| 4-6 | 14 | 7.7 |
| IC regimens |  |  |
| Taxane+ Platinum+ Fluoropyrimidine | 59 | 32.4 |
| Taxane+ Platinum | 75 | 41.2 |
| Platinum+ Fluoropyrimidine | 45 | 24.7 |
| Gemcitabine+ Cisplatin | 1 | 0.5 |
| Vinorelbine+ Nedaplatin | 1 | 0.5 |
| Paclitaxel | 1 | 0.5 |
| Response assessment |  |  |
| CR | 1 | 0.6 |
| PR | 44 | 24.2 |
| SD | 40 | 22.0 |
| PD | 11 | 6.0 |
| Not evaluated or not reported | 86 | 47.3 |

Note: Data may not add up to 100 % due to rounding.

Supplementary Table2. Comparison of Demographic and Therapeutic Characteristics of IC and Non-IC Groups in Low-risk Patients

| Characteristic | Total | Before matching | | | After propensity score matching | | |
| --- | --- | --- | --- | --- | --- | --- | --- |
|  | (n = 308), % | IC (n = 76) ，% | Non-IC (n = 232), % | *P* | IC (n = 50), % | Non-IC (n = 35), % | *P* |
| Age, y |  |  |  | 0.262 |  |  | 0.527 |
| ≤ 60 | 121 (39.3) | 34(44.7) | 87 (37.5) |  | 22 (44.0) | 13 (37.1) |  |
| ＞ 60 | 187 (60.7) | 42 (55.3) | 145(62.5) |  | 28 (56.0) | 22 (62.9) |  |
| Sex |  |  |  | 0.419 |  |  | 0.757 |
| Male | 254 (82.5) | 65 (85.5) | 189(81.5) |  | 44 (88.0) | 30 (85.7) |  |
| Female | 54 (17.5) | 11 (14.5) | 43 (18.5) |  | 6(12.0) | 5 (14.3) |  |
| Smoking history |  |  |  | 0.295 |  |  | 0.210 |
| Yes | 201 (72.3) | 54 (77.1) | 147(70.7) |  | 39 (78.0) | 23 (65.7) |  |
| No | 77 (27.7) | 16 (22.9) | 61 (29.3) |  | 11 (22.0) | 12 (34.3) |  |
| KPS |  |  |  | 0.015 |  |  | 0.300 |
| ≥90 | 181 (63.5) | 57 (75.0) | 124 (59.3) |  | 38(76.0) | 23 (65.7) |  |
| ＜90 | 104 (36.5) | 19 (25.0) | 85 (40.7) |  | 12 (24.0) | 12 (34.3) |  |
| Weight loss |  |  |  | 0.484 |  |  | 0.888 |
| Yes | 106 (35.8) | 24(32.4) | 82 (36.9) |  | 15 (30.0) | 11 (31.4) |  |
| No | 190 (64.2) | 50 (67.6) | 140 (63.1) |  | 35 (70.0) | 24 (68.6) |  |
| Pain of chest and back |  |  |  | 0.696 |  |  | 1.000 |
| Yes | 44 (14.6) | 12(16.0) | 32 (14.2) |  | 10 (20.0) | 7 (20.0) |  |
| No | 257 (85.4) | 63 (84.0) | 194 (85.8) |  | 40 (80.0) | 28 (80.0) |  |
| Tumor location |  |  |  | 0.628 |  |  | 0.854 |
| Cervical/ UT | 133 (43.2) | 31 (40.8) | 102 (44.0) |  | 21 (42.0) | 14 (40.0) |  |
| MT / LT | 175 (56.8) | 45 (59.2) | 130 (56.0) |  | 29 (58.0) | 21 (60.0) |  |
| Clinical T stage, 8th |  |  |  | 0.723 |  |  | 0.970 |
| T1-2 | 33 (11.7) | 7 (10.4) | 26 (12.0) |  | 7 (14.0) | 5 (14.3) |  |
| T3-4 | 250 (88.3) | 60 (89.6) | 190 (88.0) |  | 43 (86.0) | 30 (85.7) |  |
| Clinical N stage, 8th |  |  |  | 0.982 |  |  | 0.887 |
| N0 | 95 (33.2) | 22 (33.3) | 73 (33.2) |  | 15 (30.0) | 10 (28.6) |  |
| N1-3 | 191 (66.8) | 44 (66.7) | 147 (66.8) |  | 35 (70.0) | 25(71.4) |  |
| Clinical TNM stage, 8th |  |  |  | 0.090 |  |  | 0.503 |
| I | 11 (3.9) | 0 (0.0) | 11 (5.0) |  | 0 (0.0) | 1 (2.9) |  |
| II | 56 (19.8) | 15 (23.4) | 41 (18.7) |  | 13 (26.0) | 6 (17.1) |  |
| III | 132 (46.6) | 25 (39.1) | 107 (48.9) |  | 21(42.0) | 17 (48.6) |  |
| IV | 84 (29.7) | 24 (37.5) | 60 (27.4) |  | 16 (32.0) | 11 (31.4) |  |
| Tumor length, cm |  |  |  | 0.041 |  |  | 0.568 |
| ≤6 | 169 (58.3) | 34 (47.9) | 135 (61.6) |  | 24(48.0) | 19 (54.3) |  |
| ＞6 | 121 (41.7) | 37 (52.1) | 84 (38.4) |  | 26 (52.0) | 16 (45.7) |  |
| MLND, cm |  |  |  | - |  |  | - |
| ＜1 | 285 (100.0) | 69(100.0) | 216(100.0) |  | 47 (100.0) | 32 (100.0) |  |
| ≥1 | - | - | - |  | - | - |  |
| A[djuvant](javascript:;) [chemotherapy](javascript:;) |  |  |  | 0.011 |  |  | 0.774 |
| Yes | 120 (39.0) | 39 (51.3) | 81 (34.9) |  | 23 (46.0) | 15 (42.9) |  |
| No | 188 (61.0) | 37 (48.7) | 151 (65.1) |  | 27 (54.0) | 20 (57.1) |  |
| Radiation dose, Gy |  |  |  | 0.045 |  |  | 1.000 |
| <54 | 102 (34.3) | 18 (24.7) | 84 (37.5) |  | 10 (20.0) | 7 (20.0) |  |
| ≥54 | 195 (65.7) | 55 (75.3) | 140 (62.5) |  | 40 (80.0) | 28 (80.0) |  |
